# Multimodality CMR diagnosis of double-chambered left ventricle in a child: A case report

**DOI:** 10.1097/MD.0000000000044431

**Published:** 2025-09-26

**Authors:** Usama Anser, Lingyi Wen, Lu Zhang

**Affiliations:** a Department of Radiology, West China Second University Hospital, Sichuan University, Chengdu, People’s Republic of China.

**Keywords:** cardiac magnetic resonance, case report, congenital heart disease, double-chambered left ventricle, multimodality imaging, thrombosis

## Abstract

**Rationale:**

Double-chambered left ventricle (DCLV) is a rare congenital anomaly in which a muscular band divides the left ventricular cavity into 2 chambers. It may mimic other conditions such as left ventricular noncompaction (LVNC), thrombus, or aneurysm. This case highlights the diagnostic role of multimodality cardiac magnetic resonance (CMR) imaging.

**Patient concerns:**

We report a 12-year-old girl undergoing anthracycline chemotherapy for pre-B acute lymphoblastic leukemia who presented with non-radiating chest pain for 1 week. Her medical history included patent ductus arteriosus closure at age 2 and ongoing growth hormone therapy for short stature.

**Diagnoses:**

Diagnosis of DCLV was established based on CMR findings of a bifid apex separated by a synchronously contracting muscular band with myocardial-like signal intensity and absence of late gadolinium enhancement. A trabecular-to-compact ratio of 1.84 further excluded LVNC, and imaging features were inconsistent with diverticulum, thrombus or aneurysm.

**Interventions:**

The patient received no medical or surgical intervention. Given her stable condition and absence of concerning findings on follow-up, she remained under routine clinical observation without the need for further treatment.

**Outcomes:**

The patient remained asymptomatic during 1 year of follow-up, with no recurrence of chest pain or development of cardiac complications.

**Lessons:**

When DCLV presents with an ambiguous apical mass and abnormal cavity with complex comorbidities, it can mimic serious conditions such as LVNC, thrombus, aneurysm, or diverticulum, making accurate diagnosis challenging using echocardiography alone. In situations where echocardiographic findings are inconclusive, CMR with tissue characterization is essential to avoid misdiagnosis and unnecessary treatment. Early use of multimodality CMR in such cases helps to clarify morphology and guide clinical management.

## 1. Introduction

Double-chambered left ventricle (DCLV) is a rare congenital cardiac anomaly characterized by a muscular band that divides the left ventricle (LV) into 2 distinct chambers. Most cases are identified in childhood, often incidentally, and may present with variable symptoms ranging from chest pain and arrhythmias to incidental findings during imaging for other conditions.^[1–3]^ However, due to overlapping imaging features, DCLV is frequently misdiagnosed as other LV abnormalities such as left ventricular noncompaction (LVNC), apical thrombus, diverticulum, or aneurysm, particularly in pediatric patients.^[4–6]^

Echocardiography remains the first-line imaging modality, but its limited resolution can result in diagnostic uncertainty, especially in heavily trabeculated hearts.^[7]^ Cardiac magnetic resonance (CMR), with its superior spatial resolution, tissue characterization, and advanced techniques like four-dimensional (4D) flow, plays a critical role in confirming the diagnosis and avoiding misinterpretation.^[8,9]^

We report a case of a 12-year-old girl undergoing chemotherapy for acute lymphoblastic leukemia in whom an echogenic apical mass was initially suspected to be thrombus. CMR established the diagnosis of DCLV, preventing unnecessary intervention. This case highlights the diagnostic importance of CMR in rare structural anomalies in pediatric cardiology.

## 2. Case presentation

A 12-year-old girl was referred to the pediatric cardiology department with a 1-week history of non-radiating chest pain. Her medical history included pre-B acute lymphoblastic leukemia under treatment with anthracycline-based chemotherapy, surgical closure of a patent ductus arteriosus at age 2, and growth hormone therapy for idiopathic short stature. Genetic testing for leukemia revealed multiple mutations, including PTPN11, CEBPE-TCRA, CDKN2A, CDKN2B, and PAX5. There was no family history of congenital heart disease.

On physical examination, the patient was afebrile with heart rate of 88 beats/min, blood pressure of 112/68 mm Hg, respiratory rate of 18 breaths/min, and oxygen saturation of 99% on room air. Cardiac auscultation revealed no murmurs. Electrocardiogram (ECG) showed normal sinus rhythm without ST-T segment changes, QT prolongation, axis deviation, or conduction abnormalities. Cardiac enzyme levels (troponin T and Creatine kinase-MB) were also within normal limits.

**Figure 1.**
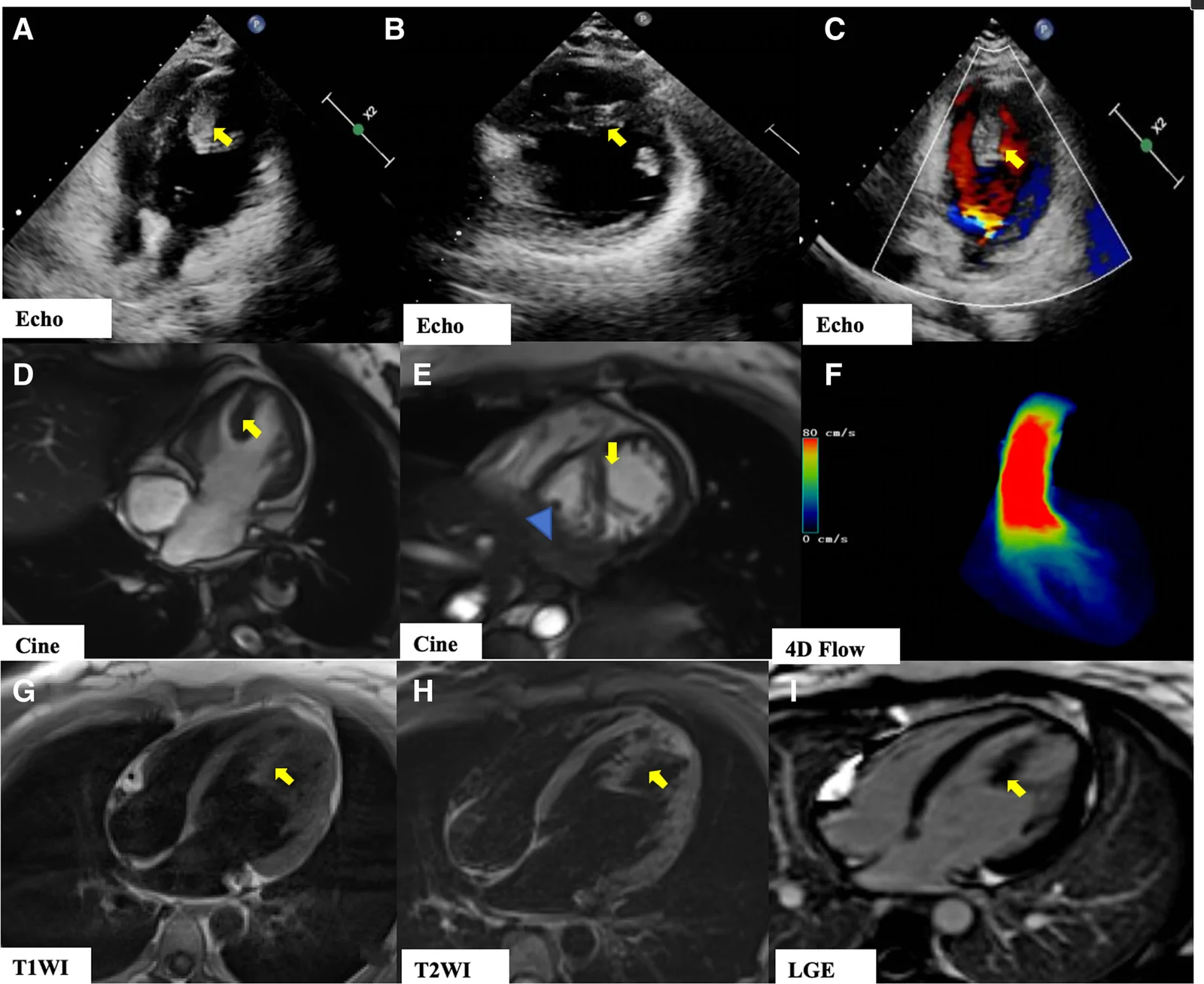
Multimodality imaging findings in a 12-year-old girl with DCLV. (A–C) Transthoracic echocardiography revealed an echogenic apical mass (yellow arrow). The short-axis view confirmed the location of the mass at the apex and its continuity with the ventricular wall (yellow arrow). Color flow Doppler ultrasound (C) showed the absence of a mosaic pattern of blood flow in the LV. (D and E) Cine CMR in the 4-chamber (D) and short-axis view (E) showed a thick muscular band (yellow arrow) dividing the apex into 2 side-by-side apical chambers. The cine short-axis view at end diastolic stage illustrated subtle increased trabeculation (E, blue arrowhead). The noncompaction-to-compact ratio was 1.84, below the threshold for left ventricular noncompaction. (F) 4D flow CMR revealed no abnormal blood flow distribution. (G–I) T1-weighted (G), T2-weighted (H), and LGE (I) images demonstrated that the muscular band (yellow arrow) exhibits signal intensity similar to that of normal myocardium. LGE showed no enhancement. CMR = cardiac magnetic resonance, DCLV = double-chambered left ventricle, 4D = four-dimensional, LGE = late gadolinium enhancement.

Transthoracic echocardiography showed an enlarged left ventricle with an echogenic apical mass measuring 4 × 3 × 4 mm, initially suspected to be a thrombus (Fig. 1A). A short-axis view confirmed the apical location and continuity of the structure with the ventricular wall (Fig. 1B). Color Doppler ultrasound did not reveal a mosaic flow pattern in the apex, suggesting preserved flow without obvious obstruction (Fig. 1C). No contrast echocardiography was performed during the evaluation. There was mild mitral regurgitation, and the left ventricular ejection fraction was estimated at 50%.

CMR revealed a bifid left ventricular apex divided by a thickened muscular band connecting the anterior and inferior apical segments, forming 2 side-by-side apical chambers (Fig. 1D and E, Supplementary Video 1). The muscular band measured approximately 8 mm in thickness and contracted synchronously with the surrounding myocardium. Cine short-axis imaging also showed mildly increased apical trabeculation (Fig. 1E, blue arrowhead). The noncompaction-to-compact ratio at end-diastole was 1.84, below the diagnostic threshold for LVNC. Signal intensity on T1- and T2-weighted images was similar to that of normal myocardium (Figs. 1G and H), and no late gadolinium enhancement was observed in the apex (Fig. 1I). Late gadolinium enhancement imaging was performed using a phase-sensitive inversion recovery sequence (TR ≈ 750 ms, TE ≈ 3.4 ms, slice thickness 8 mm) after administration of 0.1 mL/kg gadobutrol. CMR-derived left ventricular ejection fraction was 49%. 4D flow imaging demonstrated no abnormal blood flow distribution (Fig. 1F). No aneurysmal wall motion or thrombus was visualized. Given the patient’s asymptomatic status during follow-up, functional testing such as exercise ECG or stress CMR was not performed.

## 3. Discussion

Accurate diagnosis of DCLV remains challenging, especially in pediatric patients with complex comorbidities.^[1]^ In this case, DCLV was discovered in a child undergoing chemotherapy for leukemia who presented with chest pain. The initial echocardiogram raised concern for an apical thrombus, necessitating further evaluation.

Misdiagnosis of DCLV is not uncommon, particularly due to overlap with other structural abnormalities such as LVNC, LV thrombus, diverticulum, and apical aneurysms.^[4–7]^ In this patient, transthoracic echocardiography showed an echogenic mass in the apex with mildly reduced ejection fraction, but lacked contrast enhancement and definitive tissue characterization. The imaging features in CMR, including lack of wall thinning, absence of outpouching morphology, and presence of an intraventricular muscular band also helped exclude aneurysm and diverticulum.^[4,7]^

Differentiating DCLV from LVNC is particularly challenging due to the presence of apical recesses and trabeculations in both conditions. However, a trabecular-to compact myocardium ratio of < 2.3 at end-diastole (as in our case, 1.84), along with a synchronously contracting muscular band favors DCLV over LVNC.^[7]^ Thrombus typically appears noncontractile and hypointense, and aneurysms often demonstrate wall thinning or dyskinesia, none of which were present in our patient.^[6]^ To assist in similar diagnostic scenarios, we developed a comparative diagnostic table (Table 1) that summarizes key imaging distinctions between DCLV and its major differential diagnosis. The table highlights decision-support features such as contractility, signal intensity, wall motion, and trabecular-to-compact ratio. In our case, features such as synchronized myocardial contraction, trabecular ratio below 2.3, and absence of wall thinning or outpouching supported the diagnosis of DCLV over other mimics. These findings naturally raise questions about the embryologic mechanisms underlying DCLV, which remain speculative. It may result from incomplete myocardial compaction or abnormal persistence of primitive trabeculae.^[8,10]^ Experimental evidence implicates Rac1 signaling in ventricular development and cardiomyocyte polarity, suggesting that dysregulation of developmental pathways could underlie anomalies such as DCLV.^[10]^ In this case, the presence of multiple genetic mutations (PTPN11, CDKN2A/B, PAX5) in a child with leukemia may reflect a broader developmental predisposition to both oncologic and cardiac anomalies.^[11]^ Although multiple leukemia-associated mutations were identified, no cardiac-specific genetic testing was performed in this case.

**Table 1:** Comparative imaging features of DCLV and its primary differential diagnosis: LVNC, thrombus, aneurysm, and diverticulum.

Feature	DCLV	LV thrombus	Aneurysm	LVNC	Diverticulum
Morphology	Muscular band dividing apex into 2 chambers	Apical mass	Outward bulge of wall	Deep trabecular recesses	Saccular outpouching
Wall motion	Contractile	Noncontractile	Dyskinetic akinetic	Hypertrabeculated, varaible motion	Synchronous contraction
Signal intensity (T1/T2)	Isointense to myocardium	T1 hypointense; T2 hyper/mixed	Variable	Mixed	Isointense
LGE findings	No LGE at apex	Absent or rim enhancement	Bright subendocardial/ transmural LGE	Mid-wall/patchy LGE	Usually none
Trabecular/compact ratio	<2.3	–	–	>2.3	–
Communication with LV	Yes	None	Yes	N/A	Yes

DCLV = double-chambered left ventricle, LGE = late gadolinium enhancement, LV = left ventricular, LVNC = left ventricular noncompaction.

Beyond diagnosis, imaging advances continue to evolve. CMR technologies such as 4D flow, myocardial strain, and T1/T2 mapping provide enhanced structural and functional insights in pediatric cardiology.^[9]^ Although our patient remained stable over 1 year of follow-up, outcomes remain uncertain, especially in those with underlying genetic abnormalities or comorbid conditions. Continued clinical monitoring and integration of multimodal diagnostics will be essential.

## 4. Conclusion

This case highlights the diagnostic value of multimodality CMR imaging in accurately identifying DCLV and distinguishing it from mimics such as LVNC, apical thrombus, aneurysm, and diverticulum. In pediatric patients with complex clinical backgrounds and ambiguous echocardiographic findings, CMR provides critical structural and functional insights that guide clinical decision-making. Early diagnosis of DCLV through multimodality imaging helps to avoid misdiagnosis and unnecessary interventions.

## Author contributions

**Conceptualization:** Usama Anser, Lu Zhang.

**Funding acquisition:** Lingyi Wen.

**Supervision:** Lingyi Wen.

**Writing – original draft:** Usama Anser, Lu Zhang.

**Writing – review & editing:** Lu Zhang.

## References

[1] Yuan SM. Double-chambered left ventricle: clinical features comparison between children and adults. J Coll Physicians Surg Pak. 2019;29:1087–91. 10.29271/jcpsp.2019.11.1087 31659968

[2] Finocchiaro G Murphy D Pavlovic A. Unexplained double-chambered left ventricle associated with contracting right ventricular aneurysm and right atrial enlargement. Echocardiogr. 2014;31:E80–4. 10.1111/echo.12467 24299065

[3] Kato M Sasaki S Dote K. Double-chambered left ventricle with ventricular fibrillation. Intern Med. 2012;51:2245–6. 10.2169/internalmedicine.51.7972 22892513

[4] Nie W He J Lu M. A double-chambered left ventricle with a giant thrombus: a case report. Eur Heart J Case Rep. 2023;7:ytad129. 10.1093/ehjcr/ytad129 PMC10113928 37090746

[5] Zhang WM Chang DQ Huang JJ. Double-chambered left ventricle: a rare case in a child. Echocardiogr. 2019;36:192–5. 10.1111/echo.14193 30460782

[6] Malakan Rad E Awad S Hijazi ZM. Congenital left ventricular outpouchings: a systematic review of 839 cases and introduction of a novel classification after two centuries. Congenit Heart Dis. 2014;9:498–511. 10.1111/chd.12214 25159202

[7] Bao SF Wu LJ Zhang YQ Hong W-J Chen L-J Wang Q. Diagnosis of double-chambered left ventricle of superior-inferior type by echocardiography: a retrospective study of 9 subjects across two heart centers. Echocardiogr. 2021;38:169–74. 10.1111/echo.14939 33368575

[8] Destounis A Tountas C Theodosis-Georgilas A. An unusual case of double-chambered left ventricle communicated with the right ventricle through a ventricular septal defect presented during only in diastole and a concomitant mitral valve prolapse. J Echocardiogr. 2019;17:167–8. 10.1007/s12574-018-0393-5 30097833

[9] Tian J Jin D Zhu Y Liu Y. An asymptomatic double-chambered left ventricle diagnosed by contrast-enhanced ultrasound imaging: a case report. Medicine (Baltim). 2023;102:e33524. 10.1097/MD.0000000000033524 PMC10118362 37083812

[10] Leung C Lu X Liu M Feng Q. Rac1 signaling is critical to cardiomyocyte polarity and embryonic heart development. J Am Heart Assoc. 2014;3:e001271. 10.1161/JAHA.114.001271 PMC4323834 25315346

[11] Narayan P Richter F Morton S. Genetics and etiology of congenital heart disease. Curr Top Dev Biol . 2024;156:297–331. 10.1016/bs.ctdb.2024.01.009 38556426

